# Polygenic risk scores in neurological disorders: restless legs syndrome as a translational model

**DOI:** 10.1515/medgen-2026-3015

**Published:** 2026-07-08

**Authors:** Juliane Winkelmann, Barbara Schormair

**Affiliations:** Technical Universtiy Munich Institute of Human Genetics Ismaningerstrasse 22 81675 Neuherberg Germany; Helmholtz Center Munich Institute of Neurogenomics Ingolstädter Landstr. 1 85764 Neuherberg Germany

**Keywords:** polygenic risk score, restless legs syndrome, risk stratification, multi-omics, wearable sensor

## Abstract

Polygenic risk scores (PRS) aggregate the effects of common genetic variants into a single metric of disease predisposition. Many neurological disorders exhibit a polygenic architecture, thereby providing a rationale for the application of PRS in risk prediction, biological subtyping, and stratification of patients to inform clinical decision-making. Here, we use restless legs syndrome (RLS) as an informative translational model to discuss both opportunities and current constraints of PRS use in neurology. RLS has a well-characterized polygenic component with 164 GWAS risk loci, a PRS with moderate case-control discrimination (AUC 0.73) when used alone, but showing potential for higher performance (AUC 0.82–0.91) in machine-learning models incorporating non-genetic variables. We discuss how multi-omics integration, PRS-based clinical subgrouping, and rare variant penetrance modification can advance PRS development and application in RLS and contextualize these developments within the wider landscape of PRS in neurological disorders.

## Introduction

The majority of common neurological disorders are polygenic conditions in which hundreds to thousands of common genetic variants each contribute a small increment to disease susceptibility [1]. Genome-wide association studies (GWAS) have mapped this architecture with increasing resolution over two decades, and polygenic risk scores (PRS) translate the resulting summary statistics into an individual-level measure of common-variant burden by summing risk alleles weighted by their estimated effect sizes [2, 3]. In cardiology, PRS have reached the stage of clinical pilot implementation [4]. In neurology, PRS are still predominantly explored in a research context rather than clinical practice. Here, restless legs syndrome (RLS) serves as a use case to guide through for PRS application in neurological disorders. Its genetic architecture is well characterised, with 164 GWAS loci identified in 2024 [5], an estimated SNP heritability of around 20–25 %, and a substantial amount of this heritability explained by only a subset of these loci including a strong-effect anchor locus at *MEIS1* [6, 7]. PRS constructed from this data, when combined with non-genetic information through machine learning, have shown the potential to approach AUC values required for individual risk stratification [5]. Moreover, RLS possesses several quantitative phenotypes, most notably periodic limb movements during sleep (PLMS), and iron-related biomarkers, that are potentially suitable for integration into multi-modal stratification models for risk prediction or treatment guidance [8]. However, the current status in RLS also reflects important challenges of the PRS field, such as limited representation of non-European ancestries in genetic studies, variable specificity, scalability, or stage of validation of endophenotypes or biomarkers, as well as the need for large-scale datasets with comprehensive genetic and phenotypic data to develop and validate prediction models. This combination makes RLS a useful model for discussing both the promise and current limits of PRS in neurology.

We review the construction and current performance of PRS in RLS, sketch a path to improved prediction through building multi-modal risk models and clinical subgrouping, and contextualize these findings within PRS research in Parkinson’s disease (PD), Alzheimer’s disease (AD), multiple sclerosis (MS), and epilepsy.

## Polygenic risk scores: principles and construction

A PRS aggregates the weighted effects of genetic variants across the genome into a single score reflecting an individual’s genetic liability for a given trait [2]. Construction proceeds in two stages: discovery, in which variant effect sizes are estimated from GWAS summary statistics, and scoring, in which these effects are summed for each individual across genotyped or imputed variants. Methods range from simple clumping and thresholding (C+T) to Bayesian approaches such as LDpred2 and PRS-CS that model linkage disequilibrium structure and improve prediction in held-out samples [9, 10]. For diseases, PRS performance is evaluated by the area under the receiver operating characteristic curve (AUC) for case–control discrimination, odds ratios across PRS quantiles, or by pseudo-R2 measures. It depends strongly on GWAS discovery sample size, trait heritability, and the ancestry match between discovery and target populations [11].

Two general limitations warrant explicit statement. First, PRS derived from European-ancestry GWAS show substantially attenuated accuracy in non-European populations, reflecting differences in allele frequencies, linkage disequilibrium patterns, genetic architecture, and effect-size heterogeneity [11–13]. For traits where data permitted quantification, 1.6 to 4.5-fold lower genetic prediction accuracies were observed when European-trained scores are applied to other ancestries [13]. Second, even when a PRS is statistically robust, substantial overlap between the case and control score distributions often limits its use as a stand-alone diagnostic tool [3, 14]. For that reason, the more realistic near-term use cases are enrichment of high-risk groups, support for mechanistic subgrouping, and combination with imaging, fluid, physiological or clinical markers in multi-modal models for improved risk prediction and patient stratification.

## Polygenic risk scores in restless legs syndrome

### Genetic architecture of RLS

RLS, characterised by an urge to move the legs often accompanied by unpleasant sensations that emerge at rest and worsen in the evening, affects up to 10 % of older adults in European populations when defined by the IRLSSG diagnostic criteria alone, whereas clinically relevant RLS, defined by at least moderate symptom severity, a frequency of at least twice per week and associated distress or functional impairment, occurs in approximately 2 – 3 % [8, 15]. This distinction is relevant for the design and evaluation of PRS-based preventive strategies, because the absolute risk increment associated with a high PRS depends on which prevalence is used as the baseline. Family and twin studies have revealed heritability estimates of 50 – 60 % [16]. While families with Mendelian inheritance patterns have been described, earlier linkage studies as well as more recent whole exome sequencing approaches have not identified unequivocal causal variants or genes accounting for monogenic forms of RLS [16] In contrast, GWAS have been quite fruitful. The first RLS GWAS in 2007 identified three risk loci for clinically diagnosed RLS at *MEIS1*, *BTBD9* and *MAP2K5/SKOR1* [17], one of which (*BTBD9*) was independently identified in a parallel GWAS focusing on PLMS as the main phenotype [18]. Subsequent meta-analyses expanded the locus catalogue progressively to 22 loci by 2020 [6, 7]. The largest study to date from 2024 combined data from three independent GWAS (clinical-RLS cohort EU-RLS-GENE, blood-donor cohort INTERVAL and research participating customers from 23andMe (116,647 cases, 1,546,466 controls) and identified 164 independent risk loci, an eightfold increase over the previous largest study [5].

With a per-allele odds ratio (OR) of 1.82–2.16 across different studies, the association signal in the *MEIS1* gene (lead SNP rs113851554) is among the largest common-variant effects identified in GWAS of polygenic neurological disorders. Other notable examples are variants in *APOE* in Alzheimer’s disease and the *HLA-DRB1* locus in multiple sclerosis with per-allele ORs of 3 – 4. In contrast, the vast majority of common variants identified through GWAS exhibit only modest effect sizes, typically with ORs in the range of 1.05–1.2 [54–56]. *MEIS1* encodes a homeobox transcription factor involved in the regulation gene expression programs during early development, particularly in the nervous system, hematopoietic lineages, and limb structures [19]. Heterozygous Meis1 inactivation in mice produces hyperactivity at rest-phase onset, partly modelling the human symptom profile of RLS [20].

SNP-based heritability for RLS is approximately 20 % when estimated in the complete GWAS meta-analysis data [5, 6]. This meta-analysis combined individual GWAS which used different phenotype definitions and comprised different study populations. Analysing them separately showed SNP-heritabilities between 14 % and 26 %, with the highest estimate obtained in the EU-RLS-GENE dataset which included clinical RLS populations only. Interestingly, the 19 loci identified by 2017 already accounted for roughly 60 % of SNP heritability [6].

RLS prevalence differs between both sexes, with women being affected twice as often. The larger sample size of the 2024 meta-analysis allowed sex-stratified GWAS. These analyses demonstrated near-complete genetic overlap between sexes (r𝑔 = 0.96), and simulation studies suggested gene-environment interactions as the primary driver of the higher female prevalence [5].

Reflecting the overall status of the field of GWAS and PRS studies, these are largely limited to European-ancestry populations for RLS. Some other ancestries, mostly Asian, have been explored in small-scale focused candidate gene or variant studies. To date, a single GWAS has been conducted in individuals from Korea with 325 cases and 2,603 controls that yielded no genome-wide significant signals [21]. PRS performance has not been investigated outside of European-ancestry populations. Any non-European PRS application based on current summary statistics is therefore extrapolation and the expected degree of attenuation relative to European targets cannot currently be estimated from RLS data alone [12, 13].

### PRS performance and the extreme-score paradigm

PRS for RLS have been developed using both variant-count and genome-wide weighting approaches. The 2017 study constructed a weighted PRS from 20 genome-wide significant signals located in 19 risk loci and demonstrated significant risk enrichment at the score extremes: individuals in the top 0.5 % of the PRS distribution carried an OR of 17.6 (95 % CI 8.5–42.3, p = 6.9 × 10⁻²⁶) relative to the population average, and the upper versus lower quartile comparison yielded OR 5.9 (95 % CI 5.3–6.5) [6]. Distribution overlap between cases and controls remained substantial throughout the middle of the score [6]. The same trends for the discriminatory power of PRS-only approaches are consistent with what has been observed for other disorders [14, 22]. The 2024 study improved prediction by leveraging 216 genome-wide significant lead SNPs. The resulting PRS lead SNP score achieved an AUC of 0.73, outperforming a genome-wide LDpred2 score (AUC = 0.66, p = 0.0056) in this dataset. The non-genetic covariates provided a larger baseline contribution than genetic risk alone, showing a higher ΔAUC of 0.19–0.27 compared to 0.15–0.23. Combined with age, sex, and age of onset in a machine-learning framework, the overall performance achieved AUCs of 0.82–0.91 [5], representing an additional ΔAUC of 0.13 to 0.21 on top. This demonstrates the significant contribution of the PRS, providing an incremental but consistent contribution to the distributional extremes. These estimates were obtained in cross-validation designs and in hold-out samples. Further validation in external independent datasets is needed to assess their transferability and performance across more diverse cohorts. The clinical model suggested by these data is not population-wide screening but targeted enrichment: PRS identifies individuals at the distributional extremes for whom the absolute risk increment is large enough to justify prospective surveillance, early symptom monitoring and preventive intervention. Given that RLS is substantially underdiagnosed and that long-term pharmacological treatment is complicated by augmentation under dopamine agonists, which has led current clinical practice guidelines to favour alpha-2-delta ligands as first-line therapy [8, 15, 52, 53], identifying high-risk individuals before symptom onset opens the door to preventive strategies and therefore has tangible clinical value.

### Towards PRS-guided patient stratification and individualized treatment

Beyond population-level risk prediction, PRS-based identification of clinically meaningful subgroups within the diagnosed patient population remains substantially unexplored. RLS is phenotypically heterogeneous: age of onset ranges from childhood to late adulthood, severity varies from mild intermittent symptoms to severe nightly disease, the response to available treatment options such as dopamine agonists, gabapentinoids, or iron supplementation is variable, and augmentation rates under long-term dopamine agonist therapy differ substantially between individuals [8, 15]. If this clinical heterogeneity reflects in part distinct genetic subtypes, PRS analyses stratified by subphenotype could reveal different genetic architectures for early- versus late-onset disease, treatment-responsive versus treatment-nonresponsive patients, or for augmentation-prone versus augmentation-resistant patients.

In principle, a PRS trained on early-onset cases could identify a biologically distinct subgroup for whom the mechanistic target is more clearly defined. Treatment selection, particularly the choice between dopamine agonists, alpha-2-delta ligands and iron therapy, could in future be guided by genetic profile. Pathway-specific PRS, analogous to the lysosomal and immune pathway scores constructed for PD [23], represent a natural methodological extension: scores focused on dopaminergic, glutamatergic and iron homeostasis loci could be constructed separately and tested for differential associations with clinical response outcomes. The subgrouping agenda requires future prospective cohort data with standardised phenotyping, treatment records and long-term follow-up.

Integrating complementary omics layers into multi-modal risk models offers a further avenue for refining individual risk prediction. The predictive ceiling of the PRS as a purely genotype-based measure is set by the SNP heritability of the trait, approximately 20 % for RLS. Based on the observed impact of non-genetic factors in the machine-learning risk prediction models for RLS as well as the suggested gene-environment interactions playing a role in the higher female prevalence, multi-modal prediction models integrating diverse data layers are a promising approach in RLS.

Expression quantitative trait locus (eQTL) data offer a first extension. The 2024 RLS GWAS used cis-eQTL annotation to prioritise candidate effector genes at several loci including glutamate receptors *GRIA1* and *GRIA4*, both targets of existing anticonvulsants [5]. Extension to a transcriptomic risk score (TRS), in which observed blood or tissue gene expression levels rather than genotypes serve as predictors, could capture both genetic regulation (via cis-eQTL effects partly shared with the PRS) and non-genetic modulators of transcript abundance, including environmental exposures, cell-type composition, medication and ageing. By incorporating these dynamic contributions, a TRS could in principle explain a larger fraction of phenotypic variance than genotype alone.

Plasma proteomics represent a second layer with immediate clinical relevance. Iron metabolism parameters, including ferritin, transferrin saturation and soluble transferrin receptor, are established disease modifiers and are routinely measured in clinical practice [8]. Their systematic inclusion in composite prediction models is supported by observational data but has not been evaluated in a prospective PRS-stratified design.

Epigenomic data constitute a third layer. Epigenome-wide association studies in RLS have developed first epigenetic risk scores based on methylation profiles in blood and selected brain tissue samples, but their predictive power as a single-modality-score was limited and evaluation in combined models has not been performed [24, 25]. Finally, Metabolomics presents a fourth layer that is largely unexplored in RLS, so far. However, the causal relationship between RLS and type 2 diabetes suggested by Mendelian randomisation analyses [5] could point towards shared metabolic dysregulation potentially detectable at the metabolite level before clinical disease onset. The integration of these layers within machine-learning frameworks trained on biobank-scale data, with nested cross-validation to prevent overfitting, represents the next methodological frontier for RLS risk prediction.

### Wearables and polysomnography as RLS phenotyping layers

A methodological advantage of RLS for PRS-based research is the availability of an objective, quantifiable endophenotype that extends beyond symptom-based diagnosis and binary case–control classification. Periodic limb movements during sleep (PLMS), present in approximately 80 % of RLS patients, are measurable by polysomnography (PSG) and provide a continuous neurophysiological outcome variable with established inter-laboratory reliability [8, 15]. PSG-derived PLMS indices, particularly the periodic limb movement index (PLMI), have been used as continuous outcome measures in published cohort studies [26, 27]. These observations support the use of PLMS as a quantitative intermediate phenotype for PRS validation and multimodal modelling, although the use of PLMS as a primary endpoint in PRS-stratified prospective studies has not yet been reported.

In principle, research and consumer-grade accelerometry offers a scalable complement to PSG. At present, evidence supports lower-limb actigraphy, rather than consumer-grade wrist-worn accelerometry. In a recent validation study, leg actigraphy with the SOMNOwatch system achieved a sensitivity of 86.7 % and a specificity of 92.3 % for PLMS detection against PSG, supporting its potential use for research phenotyping in larger cohorts [28]. Large-scale resources such as the UK Biobank accelerometry substudy, which includes 7-day wrist-accelerometry recordings in more than 100,000 participants, allow to study accelerometer-derived movement or sleep abnormalities associated with genetic liability. This concept is indirectly supported by prior UK Biobank work showing that accelerometer-derived sleep traits capture signals at known RLS loci, including *MEIS1* and *BTBD9* [29]. The German National Cohort (NAKO Gesundheitsstudie), with a large accelerometry substudy nested in its more than 200,000 participants, represents a comparable European resource that could in principle support analogous PRS-stratified analyses of movement and sleep phenotypes. PRS-stratified subgroup analyses in such datasets are therefore methodologically plausible and subclinical RLS-related motor abnormalities could be investigated in PRS-defined groups. Longitudinal electronic health record data, including iron supplementation prescription histories, dopaminergic medication trajectories and comorbidity accumulation patterns, further enrich such designs.

The integration of quantitative PSG-derived PLMS measures, lower-limb wearable proxy measures, and genomic risk information is of interest in RLS because it links inherited susceptibility to an objective intermediate phenotype that can be followed longitudinally. It provides a useful translational framework in a condition that is otherwise largely defined by subjective symptoms. Such a data structure could support longitudinal mixed-effects or machine-learning models that test how genetic risk is expressed as progressive endophenotypic change during preclinical and early symptomatic phases.

## PRS in other neurological disorders: a comparative perspective

The following subsections summarise PRS performance and translational utility for four neurological conditions that differ from RLS in genetic architecture, available biomarkers and clinical context. A consistent pattern across all five disorders is that PRS derived from large European-ancestry GWAS achieve moderate-to-good case-control discrimination, are routinely validated in European populations but not in non-European populations, and that clinical utility of PRS alone is limited relative to combined models.

### Parkinson disease

PD has a well-characterised polygenic component alongside well-known monogenic forms. The 2019 meta-analysis by Nalls et al. identified 90 independent risk variants across 78 GWAS loci [30]. A large-scale multi-ancestry GWAS for PD has been conducted in European, East Asian, Latin American, and African ancestry group and identified 78 genome-wide significant loci, including 12 potentially novel loci [31]. PRS for PD achieve AUCs of 0.62–0.69 for case–control discrimination and replicated across UK Biobank, FinnGen and multiple European clinical cohorts [32, 33]. Beyond disease risk, PRS have been associated with earlier age at onset and, in some but not all cohorts, faster cognitive and motor decline, with inconsistency likely reflecting clinical heterogeneity within the PD diagnosis [32, 34].

The most productive PRS development in PD has been biological pathway stratification. A lysosomal pathway PRS predicts cognitive decline more accurately than the overall disease-risk PRS [34], illustrating that the genetic heterogeneity within PD can be partially resolved by constructing scores around specific biological mechanisms. Immune pathway PRS similarly show differential associations with clinical subtypes [23]. This pathway-specific approach is directly transferable to RLS, where dopaminergic, glutamatergic and iron homeostasis loci could anchor separate sub-scores. PRS have also been applied in PD prodromal cohorts, where higher scores associate with a greater probability of meeting the Movement Disorder Society (MDS) research criteria for prodromal Parkinson’s disease, which combine age-adjusted risk and prodromal markers (including REM sleep behaviour disorder, hyposmia, autonomic dysfunction, and subtle motor signs) into a probability estimate of underlying neurodegeneration [35], [51], suggesting that genetic risk stratification could support earlier intervention trials.

### Alzheimer disease

AD genetics is dominated by the *APOE* locus, where the ε4 allele confers a three- to tenfold risk increase depending on zygosity. Beyond *APOE*, a 2022 GWAS identified over 80 loci enriched in microglial and immune pathways [36]. AD PRS including *APOE* achieve AUCs of 0.62–0.75, replicated in ADNI, BioFINDER and EPAD for conversion from mild cognitive impairment to dementia [37]. PRS performance benchmarking showed that models integrating *APOE* separately from the remaining PRS reached higher AUCs compared to including *APOE* in the PRS [38]. The most clinically relevant advance has been the combination of AD PRS with fluid biomarkers: combining the PRS with CSF or plasma amyloid and tau measurements improves prognostic accuracy substantially and enables finer stratification of individuals at the pre-dementia stage [39]. This biomarker-plus-PRS model is directly analogous to the iron-biomarker-plus-PRS framework proposed for RLS, and the AD experience suggests that the incremental value of adding molecular biomarkers to a PRS is substantial and clinically actionable.

### Multiple sclerosis

MS has the largest GWAS locus catalogue of the disorders reviewed here, with over 200 susceptibility loci, the HLA region contributing a disproportionate share of genetic risk [40]. MS PRS achieve AUCs of 0.73–0.80 in European-ancestry cohorts, and individuals in the top 10 % of the distribution carry a 5- to 15-fold elevated risk relative to the population median [41]. Replication is well established across Swedish, UK and Dutch birth-cohort studies [42].

MS represents the strongest current candidate among neurological conditions for PRS-guided clinical surveillance. The rationale is straightforward: highly effective disease-modifying therapies exist whose benefit is greatest when initiated early in the disease course, before irreversible neurological damage accumulates. PRS could identify high-risk first-degree relatives of affected patients for targeted monitoring with MRI and cerebrospinal fluid biomarkers, allowing treatment initiation at the earliest radiologically isolated or clinically isolated syndrome stage. This application is proposed based on existing PRS performance data. So far, prospective surveillance trials using PRS selection criteria have not been reported. Non-European studies remain sparse, limiting current applicability outside European-ancestry populations.

### Epilepsy: from risk prediction to treatment guidance

Epilepsy is the neurological condition for which the translational argument for PRS moves most directly from risk prediction to clinical decision-making. An ILAE Consortium GWAS identified 26 risk loci, with markedly different genetic architectures for genetic generalised epilepsy (GGE; SNP heritability 30–40 %) and non-acquired focal epilepsy (NAFE; SNP heritability 9 – 16 %) [43]. This architectural distinction has direct clinical implications, because GGE and NAFE require different antiseizure medications, and misclassification at the time of a first unspecified seizure event is common.

A FinnGen analysis by Heyne et al. provides an excellent example of PRS with potential for informing a treatment decision: individuals with a GGE PRS above two standard deviations had a 37 % probability of developing GGE within ten years of an initial seizure, compared with 5.6 % for those below –2 standard deviations [44]. This difference is clinically relevant because the decision to initiate antiseizure medication after a first event, and the choice of agent, depends substantially on the probability that the event represents the first manifestation of a defined epilepsy syndrome. A high GGE PRS at first presentation would support early initiation of broad-spectrum antiseizure medication and discourage the use of sodium channel blockers, which may exacerbate absence and myoclonic components of GGE. This application uses PRS to inform medication selection rather than simply quantify risk, and is among the most concrete clinical use cases reviewed here. The FinnGen finding has not yet been externally replicated and should be treated as provisional.

## Rare variants and polygenic background: a brief note

The integration of common-variant PRS with high-impact rare variants is an emerging methodological approach with direct relevance to RLS. Evidence for a joint contribution of both types of genetic variation to disease risk and clinical expression in neurological disorders is most advanced for Parkinson’s disease and epilepsy. In Parkinson’s disease, penetrance among carriers of LRRK2 p.G2019S is modified by polygenic background, indicating that common-variant burden contributes to whether a carrier develops disease [45]. Similar observations have now been reported for GBA1-associated Parkinson’s disease, in which both variant severity and Parkinson’s disease PRS influence carrier risk [46]. In epilepsy, polygenic background has been associated with penetrance and phenotypic expression in families carrying rare variants linked to monogenic epilepsies [47], and common epilepsy risk variants are enriched in multiplex families that had previously been investigated for rare monogenic causes [48]. Together, these findings support a model in which rare variants establish a major baseline susceptibility, while common-variant burden shifts penetrance, severity, age at onset, or specific phenotypic expression.

In RLS, rare coding variants in *MEIS1* have been identified in familial RLS and functionally implicated in disease biology, including the missense variant MEIS1 p.R272H, which was also reported in a familial RLS case series with an unaffected carrier, consistent with reduced penetrance [49, 50]. These variants are plausible first targets for PRS-by-rare-variant analyses in RLS.

## Conclusion and outlook

Three research priorities are clear for restless legs syndrome. First, non-European GWAS are urgently needed. The complete absence of non-European RLS replication data is both a scientific limitation and an equity concern, and trans-ancestry meta-analysis must be a central goal of the next wave of RLS genetic studies. Second, prospective PRS-stratified cohort studies should be established, incorporating wearable-derived continuous phenotyping and longitudinal iron biomarker monitoring, to test whether genetic risk stratification translates into earlier diagnosis, targeted prevention and improved treatment outcomes. Third, PRS-based clinical subgrouping, informed by pathway-specific scores and multi-omics data, should be pursued to determine whether genetic subtypes within the RLS diagnosis predict differential treatment responses.

The comparative evidence from PD, AD, MS and epilepsy confirms that the value of PRS in neurology extends well beyond simple risk prediction. In epilepsy, PRS can for example inform the decision to treat after a first seizure and guide medication choice. In MS, PRS can stratify first-degree relatives for surveillance, and in PD, pathway-specific scores dissect clinical heterogeneity. RLS, with its well-defined genetic signals, modifiable risk factors, objective endophenotypes and substantial unmet clinical need, provides a suitable translational model for this broader effort.
